# Effects of yogurt as a deglutition aid on disintegration and dissolution of oral tablets

**DOI:** 10.1111/jtxs.12665

**Published:** 2022-02-19

**Authors:** Taisuke Matsuo, Yoshiyuki Tabata, Hina Sasaki, Yuki Yoshida, Yayoi Gotoh, Toshio Suzuki, Michiko Obara, Yasuyuki Sadzuka, Takashi Tomita

**Affiliations:** ^1^ Division of Advanced Pharmaceutics, Department of Clinical Pharmaceutical Sciences School of Pharmacy, Iwate Medical University Shiwa‐gun Japan; ^2^ Research and Development Laboratories Fujicco Co., Ltd. Chuo‐ku Japan; ^3^ Department of Pharmaceutical Sciences, Faculty of Pharmaceutical Sciences Teikyo Heisei University Nakano‐ku Japan

**Keywords:** deglutition aid jelly, disintegration, disorder, dissolution, xanthan‐gum based food thickening agent, yogurt

## Abstract

Patients with dysphagia have difficulty swallowing oral medications. Swallowing aid foods, such as deglutition aid jellies and food thickeners, are often used to help such patients take oral medications. Yogurt is occasionally used to help swallow medications. It is also advantageous as it is nutritious and easy to swallow. However, the influence of yogurt on the pharmacokinetics of oral medications is poorly understood. In this study, we aimed to evaluate yogurt as a potential swallowing aid for the intake of oral tablets, by comparing the physical properties and effects of yogurt on disintegration and dissolution profiles of various oral tablets with deglutition aid jelly and xanthan gum‐based food thickener. Yogurt and the food thickener were found to extend the disintegration time of several tablets; however, this increase was unremarkable. Although dissolution of magnesium oxide tablets decreased by 6%, 14%, and 25% after immersion in deglutition aid jelly, food thickener, and yogurt, respectively, at 15 min, this impact on dissolution reduced over time (dissolution rates of all samples at 120 min were over 90%). Rheological measurements showed that yogurt and food thickeners have a weak gel structure and therefore have better fluidity than deglutition aid jelly. The adhesiveness and dynamic viscosity of yogurt were higher than those of the food thickener, which delayed tablet disintegration and reduced the dissolution rate. However, these effects were not substantial. We can thus conclude that yogurt may be a useful swallowing aid for patients with deglutition disorders who take oral medications.

## 1 INTRODUCTION

As a result of an aging society, the number of people with dysphagia has increased. Aging and disease can make swallowing difficult for many patients, which increases the risk of accidental aspiration of food and drinks (Feng et al., 2013; Fujitani, 2000; Iida et al., 2013; Oad et al., 2019), and also makes it difficult for patients to take oral medications (Kelly et al., 2009; Oad et al., 2019). To aid medication intake, patients with dysphagia use food thickeners to increase the viscosity of liquids, and deglutition aid jellies to convert solids into a gelatinous form (Ookoshi, 2004). However, these swallowing aids can interfere with the disintegration of dosage forms and the dissolution of the drugs within these dosage forms (Cichero, 2013; Morita et al., 2011; Matsuo et al., 2020; Obara et al., 2020; Tomita, Goto, et al., 2019). Immersing tablets in food thickeners for a long time can delay or inhibit tablet disintegration (Matsuo et al., 2020). Therefore, it is important to minimize immersion time (≤1 min) to avoid problems with disintegration (Matsuo et al., 2020). The immersion of voglibose orally disintegrating (OD) tablets in deglutition aid jelly for 10 min has been reported to reduce the pharmacological activity of voglibose (Tomita, Goto, et al., 2019). Research on the effects of food thickeners and deglutition aid jellies on oral medications has increased our understanding of this subject.

Yogurt is rich in nutrients, such as proteins, minerals, and vitamins, and it has been reported to lower the risk of sarcopenia (Cuesta‐Triana, et al., 2019; Hashemi Gahruie et al., 2015). Yogurt has moderate viscosity and hardness, making it a suitable training meal for patients with dysphagia. The use of yogurt to facilitate swallowing of medications was recommended by the Japanese Society of Dysphagia Rehabilitation Dysphagia Diet Committee (DDC) in the Japanese Dysphagia Diet 2013 (Fujitani et al., 2013). In clinical practice, yogurt is used to help patients with dysphagia take medications (Fujitani, 2000). It is also used as an index of hardness for foods (Ookoshi, 2004). However, the effects of yogurt on the disintegration and dissolution of oral medications are poorly understood.

The properties of yogurts vary depending on the lactic acid bacteria used in manufacture, and these properties affect the degree of swallowing required. Some lactic acid bacteria synthesize exopolysaccharides, which affect the viscosity, hardness, and adhesiveness of yogurt. *Lactococcus lactis* subsp. *cremoris* FC (*L. cremoris* FC) is a strain of lactic acid bacteria isolated from fermented milk from the Caucasus region (Ishida et al., 2005). The strain *L. cremoris* FC produces exopolysaccharides that affect the texture and stability of the yogurt (De Vuyst & Degeest, 1999; Ishida et al., 2005; Ruas‐Madiedo & de los Reyes‐Gavilán, 2005). It has been reported that yogurt manufactured using *L. cremoris* FC decreases the rate of laryngeal invasion and aspiration in patients with dysphagia, compared with yogurt manufactured using other strains of lactic acid bacteria (Gotoh et al., 2019). In this study, we aimed to assess yogurt as a potential swallowing aid for oral tablets. This was achieved by comparing the effects of yogurt on the disintegration and dissolution of magnesium oxide tablets with those of the xanthan‐based food thickener and deglutition aid jelly. We chose magnesium oxide tablets as these are commonly used by patients in care facilities. In addition, we evaluated the effects of these swallowing aids on other oral medications, including film‐coated tablets, sugar‐coated tablets, enteric‐coated tablets, and OD tablets. The physical properties, adhesiveness, dynamic viscosity, and rheology of the swallowing aids were also compared. We hypothesized that yogurt manufactured using *L. cremoris* FC could be used as an aid for the administration of oral medication.

## 2 MATERIALS AND METHODS

### 2.1 Materials

**FIGURE 1:**
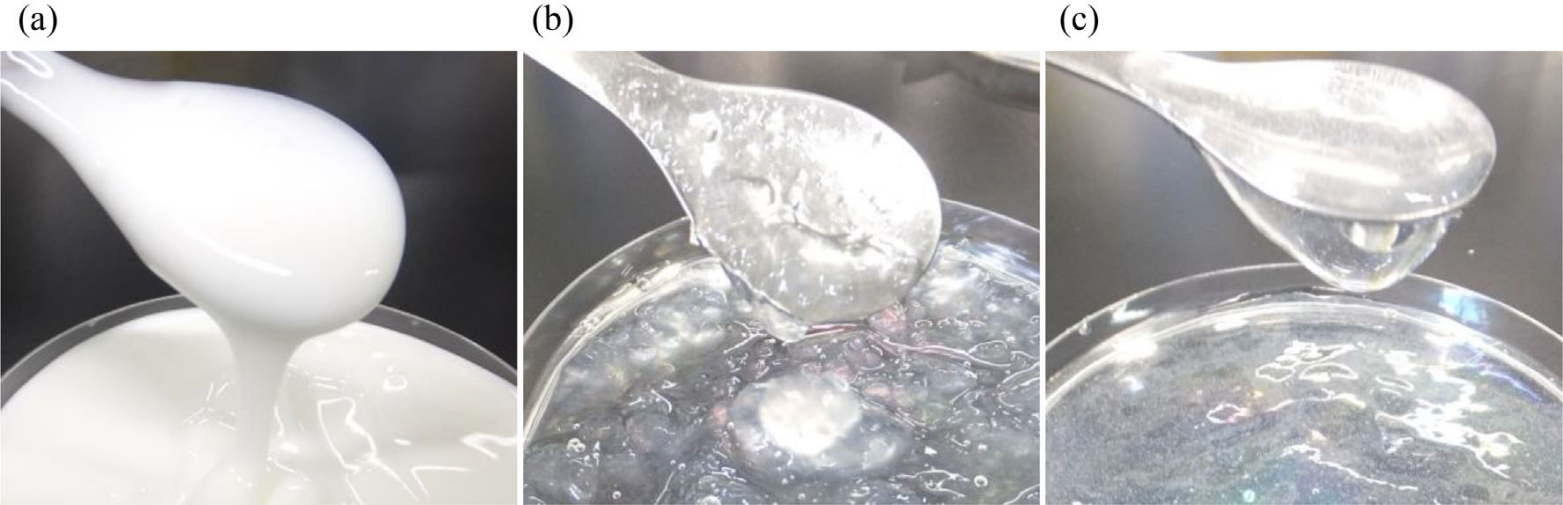
Appearances of swallowing aid foods. Appearances of (a) yogurt, (b) deglutition aid jelly, and (c) food thickener are shown

The choice of medications for use in this study was based on the results of a questionnaire survey conducted in care facilities and a previous study on the effects of food thickeners on disintegration and dissolution of medications (Matsuo et al., 2020, 2021; Tomita, Sakai, et al., 2019). Details of the medications used in this study are shown in Table 1. Deglutition aid jelly, xanthan gum‐based food thickener, and yogurt made with *L. cremoris* FC were purchased from Ryukakusan Co. Ltd. (Swallowing aid jelly; Tokyo, Japan), Clinico Co. Ltd. (Tsururinko Quickly, 3.0 g/pack; Tokyo, Japan), and Fujicco Co. Ltd. (Caspian Sea Yogurt; Hyogo, Japan), respectively. The appearances of these and their ingredients are shown in Figure 1 and Table 2, respectively.

**TABLE 1:** Medications

General name	Product name	Company	Lot number	Characteristic
Magnesium oxide	Magmitt® table 330 mg	Kyowa Chemical Industry Co., Ltd.	19B028	Naked tablet
	Magnesium oxide tablet 330 mg “Yoshida”	Yoshida Pharmaceutical Co., Ltd.	B775	Naked tablet
	Magnesium oxide tablet 330 mg “MOCHIDA”	Mochida Pharmaceutical Co., Ltd.	KE02	Naked tablet
	Magnesium oxide tablet 330 mg “KENEI”	Kenei Pharmaceutical Co., Ltd.	919,709	Naked tablet
	Magnesium oxide tablet 330 mg “Mylan”	Mylan Inc.	M238AB7	Naked tablet
Furosemide	Lasix® tablet 40 mg	Sanofi K.K.	0K198A	Naked tablet
	Furosemide table 40 mg “TAKEDA TEVA”	Teva Takeda Pharma Ltd.	F21123	Film coated tablet
	Furosemide tablet 40 mg“NP”	Nipro Corpotation	20G131	Naked tablet
Amlodipine	Norvasc® OD tablet 2.5 mg	Pfizer Japan Inc.	DC7610	OD tablets
	Amlodin® OD 2.5 mg	Sumitomo Dainippon Pharma Co., Ltd.	3211C	OD tablets
	Amlodipine OD 2.5 mg “SAWAI”	Sawai Pharmaceutical Co., Ltd.	620,201	OD tablets
	Amlodipine 2.5 mg “SAWAI”	Sawai Pharmaceutical Co., Ltd.	119,406	Film coated tablet
Aspirin	Bayaspirin tablets 100 mg	Bayer Yakuhin, Ltd.	JPS3573	Enteric coated tablet
Sodium valproate	Valerin® 200 mg	Sumitomo Dainippon pharma Co., Ltd.	2516C	Sugar coated tablet

**TABLE 2:** Ingredients of swallowing aid foods

Yogurt	Deglutition aid jelly	Food thickener
Raw milk	Erythritol	Dextrin/xanthan gum
	Reduced malt sugar syrup	Calcium lactate
	Agar/gelling agent (polysaccharide thickener)	Trisodium citrate
	Acidulant	
	Calcium lactate	
	Flavor	
	Sweetener (stevia)	
	Gardenia pigment	

### 2.2 Preparation of food thickener

The method for the preparation of the xanthan gum‐based food thickener has been previously described (Matsuo et al., 2020). In brief, xanthan gum‐based food thickener (3.0 g) was added to 100 ml of soft water (Suntory Tennensui; Suntory Beverage & Food Limited, Japan) and mixed. The prepared food thickener was used 2 min after mixing.

### 2.3 Disintegration test

The disintegration test was performed as described in the Japanese Pharmacopeia 17th Edition (The Ministry of Health, Labour and Welfare of Japan, 2016a). This method has previously been described in the literature (Matsuo et al., 2020, 2021). The tablets were immersed in each deglutition aid for 1 min, and then transferred to a basket of the disintegration testing apparatus (NT‐40HS, Toyama Sangyo Co., Ltd., Japan). Approximately 0.5–1.0 g of each of the swallowing aids was left attached to the tablets. The test was carried out using the first fluid (sodium chloride (2.0 g) and hydrochloric acid (7.0 ml) in 1000 ml of water, pH 1.2) and the second fluid (0.2 mol/L potassium dihydrogen phosphate (250 ml) and 0.2 mol/L sodium hydroxide (118 ml) in 1000 ml of water, pH 6.8). The enteric‐coated tablets were tested in both fluids, while the other tablets were tested only at pH 1.2. The disintegration time was defined as the time taken for the contents of the tablets to be completely released from the baskets. The maximum test time was 2 h. Each experiment was performed using nine tablets.

### 2.4 Dissolution test for magnesium oxide tablets

The dissolution test was performed using the paddle apparatus, as described in the Japanese Pharmacopeia 17th Edition (The Ministry of Health, Labour and Welfare of Japan, 2016b). This method has previously been described in the literature (Matsuo et al., 2021). Magnesium oxide tablets were pre‐treated with the swallowing aid foods, as described in Section 2.3. Magnesium oxide content in the dissolution medium was measured by chelate titration using ethylenediaminetetraacetic acid (EDTA):

1mlof0.05mol/LEDTA=2.015mgof magnesium oxide.

### 2.5 Adhesiveness

The evaluation of adhesiveness has previously been described in the literature (Matsuo et al., 2020). In brief, the deglutition aids (10.0 g) were added to 100 ml polyvinylpyrrolidone (PVP) or glass beakers. Each beaker was reversed for 1 min and adhesiveness was defined as the residue rate in the beaker.

### 2.6 Rheological measurements

The yogurt was tested at 10 and 20°C, while the other samples were tested at 20°C. Measurements were obtained using a controlled stress rheometer (MCR‐102, Anton Paar, Austria) equipped with cone plates (50 mm diameter, angle 1°). The temperature of the measuring cell was maintained with a Peltier system (P‐PTD200, Anton Paar, Austria). The samples were placed between a cone plate and the foundation support of the rheometer. For dynamic viscosity measurements, the shear rate was increased from 20 to 50 s^−1^ within 1 min. Next, dynamic viscoelasticity measurements were performed. To determine the linear viscoelastic region, strain‐dependent measurements were made from 0.01% to 10% strain, at a frequency of 1 Hz. As a result, 0.1% strain was adopted as the measurement condition. To evaluate the viscoelastic properties of the samples, frequency‐dependent measurements were made between 0.1 and 10 Hz at 0.1% strain, and the storage modulus (*G'*), loss modulus (*G"*) were determined.

### 2.7 Statistical analysis

Data are presented as mean ± standard deviation. Statistical analyses were performed using Student's *t*‐test or one‐way analysis of variance with post hoc test (Dunnett's test or Tukey–Kramer method). Differences were considered statistically significant at *p* < 0.05.

## 3 RESULTS

### 3.1 Effects of yogurt on disintegration and dissolution of magnesium oxide tablets

**FIGURE 2:**
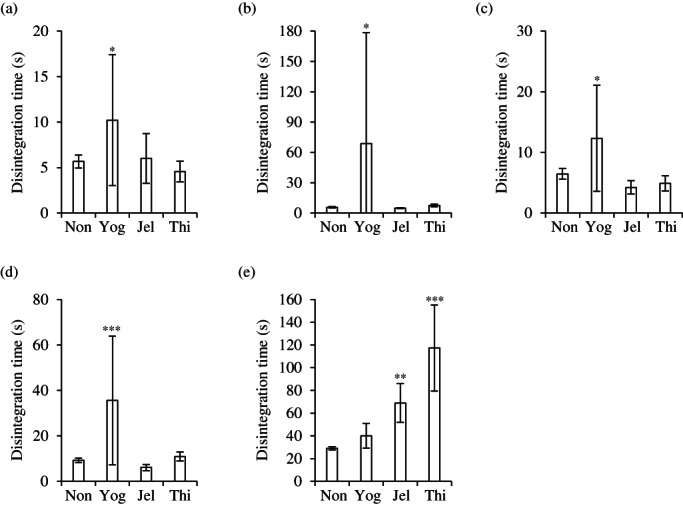
Disintegration time of magnesium oxide tablets. Five brands of magnesium oxide tablets, (a) Magmitt®, (b) “MOCHIDA,” (c) “KENEI,” (d) “Yoshida,” and (e) “Mylan” were immersed in swallowing aids for 1 min. All experiments were performed in an aqueous medium of pH 1.2 (*n* = 9). Jel, immersed in deglutition aid jelly; Non, not immersed; Thi, immersed in food thickener; Yog, immersed in yogurt. *p < 0.05; **p < 0.01, and ***p < 0.001, which represent significant differences (compared with non‐immersed tablets, Dunnett's test)

**FIGURE 3:**
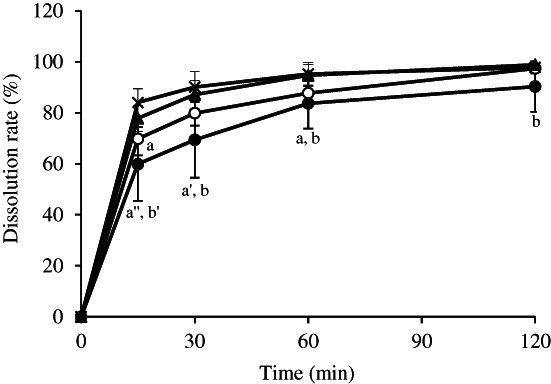
Dissolution rate of Magmitt® tablets after immersion in swallowing aid foods. The dissolution rates of Magmitt® tablets were measured at 15, 30, 60, and 120 min by chelate titration (*n* = 3). The symbols in the figure indicate: × not immersed, ● yogurt, and ▲ deglutition aid jelly, 〇 food thickener. ^a^ *p* < 0.05; ^a’^ *p* < 0.01; and ^a”^ *p* < 0.001 (for immersed tablets vs. non‐immersed tablets). ^b^ *p* < 0.05 and ^b’^ *p* < 0.01 (for tablets immersed in yogurt vs. tablets immersed in deglutition aid jelly) which represent significant differences (compared with all samples tested at the same time points, Turkey Kramer test)

The disintegration times of magnesium oxide tablets immersed in yogurt were compared with the disintegration times in deglutition aid jelly and food thickener (Figure 2). Magmitt® tablets, and the magnesium oxide tablets, “MOCHIDA,” “KENEI,” and “Yoshida,” disintegrated within 4–11 s, even after immersion in deglutition aid jelly and food thickener. The disintegration times of the tablets immersed in yogurt reached a maximum of 70 s. The magnesium oxide tablet “Mylan” disintegrated in approximately 30 s without immersion in any deglutition aid. When the tablets were immersed in deglutition aid jelly, food thickener, and yogurt, their disintegration times were approximately 1 min, 2 min, and 40 s, respectively. Parts of the swallowing aids were left attached to tablets after disintegration, which might have affected dissolution. The dissolution rate of the Magmitt® tablets was measured (Figure 3). The dissolution rates of magnesium oxide without the deglutition aid foods at 15, 30, 60, and 120 min were 84%, 90%, 95%, and 98%, respectively. The dissolution rates after immersion in yogurt, food thickener, and deglutition aid jelly at 15 min were 60%, 70%, and 78%, respectively. However, dissolution rates of all the samples at 120 min were over 90%, and were similar to the dissolution rates of the non‐immersed tablets.

### 3.2 Effects of yogurt on the disintegration of other oral tablets

**FIGURE 4:**
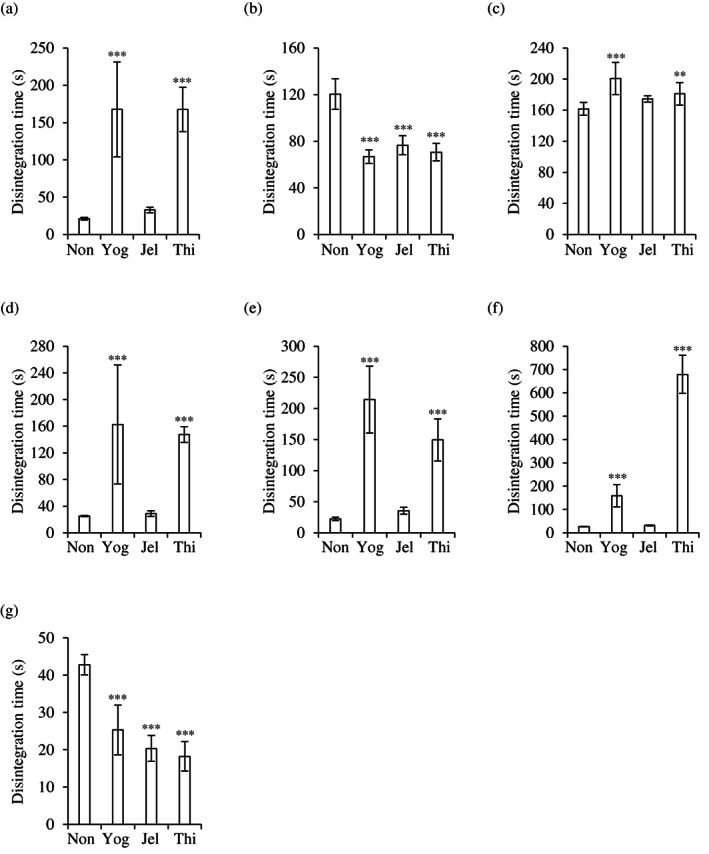
Disintegration times of furosemide and amlodipine tablets. (a) Lasix®, (b) furosemide “TAKEDA TEVA,” (c) “NP,” (d) Norvasc® OD, (e) amlodine® OD, (f) amlodipine OD “SAWAI,” and (g) amlodipine 2.5 mg “SAWAI,” tablets were immersed in swallowing aid foods for 1 min before disintegration time experiments were conducted. All experiments were performed in aqueous medium of pH 1.2 (*n* = 9). Jel, immersed in deglutition aid jelly; Non, not immersed; Thi, immersed in food thickener; Yog, immersed in yogurt. **p < 0.01 and ***p < 0.001, which represent significant differences (compared with non‐immersed tablets, Dunnett's test)

**FIGURE 5:**
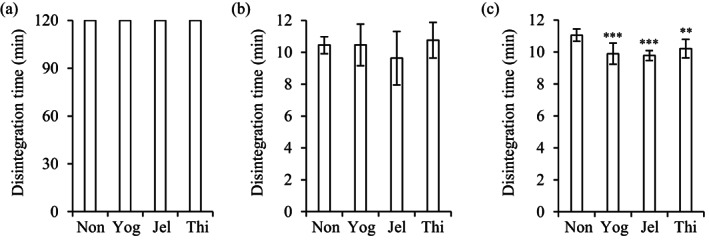
Disintegration times of aspirin and sodium valproate tablets. The tablets were immersed in swallowing aid foods for 1 min. The disintegration of Bayaspirin tablets was evaluated at (a) pH 1.2, and (b) pH 6.8 (*n* = 9). (c) The disintegration of sodium valproate tablets was evaluated at pH 1.2 (*n* = 9). Jel, immersed in deglutition aid jelly; Non, not immersed; Thi, immersed in food thickener; Yog, immersed in yogurt. **p < 0.01 and ***p < 0.001, which represent significant differences (compared with non‐immersed tablets, Dunnett's test)

Three furosemide tablets (two uncoated and one film‐coated tablet), four amlodipine tablets (one film‐coated and three OD tablets), an aspirin tablet (enteric‐coated), and a sodium valproate tablet (sugar‐coated) were evaluated (Figures 4 and 5). Immersion of these tablets in deglutition aid jelly did not prolong the disintegration time of any of the tablets, compared with non‐immersed tablets. Yogurt and food thickener delayed the disintegration of Lasix® (furosemide) tablets and three amlodipine OD tablets (Figure 4). In contrast, the film‐coated amlodipine tablets showed rapid disintegration after immersion in all the deglutition aids (Figure 4). The enteric‐coated aspirin tablets did not disintegrate in the first test medium (pH 1.2) in any of the cases, but disintegrated in approximately 10 min in the second test medium (pH 6.8) (Figure 5). The disintegration of the sugar‐coated Valerin® (sodium valproate) tablets was not affected by immersion in any of the deglutition aids.

### 3.3 Comparison of physical properties of the deglutition aids

**FIGURE 6:**
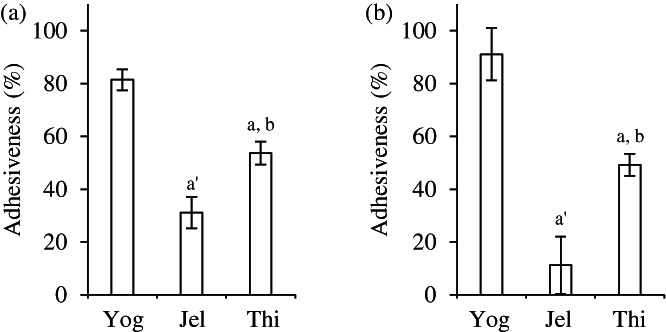
Evaluation of adhesiveness. Adhesiveness of yogurt (Yog), deglutition aid jelly (Jel), and food thickener (Thi) were compared (*n* = 3). (a) adhesiveness in glass beakers, (b) adhesiveness in polyvinylpyrrolidone (PVP) beakers. ^a^ *p* < 0.01; ^a’^ *p* < 0.001 (vs. yogurt), ^b^ *p* < 0.01 (vs. deglutition aid jelly) represent significant differences (Turkey–Kramer test)

**FIGURE 7:**
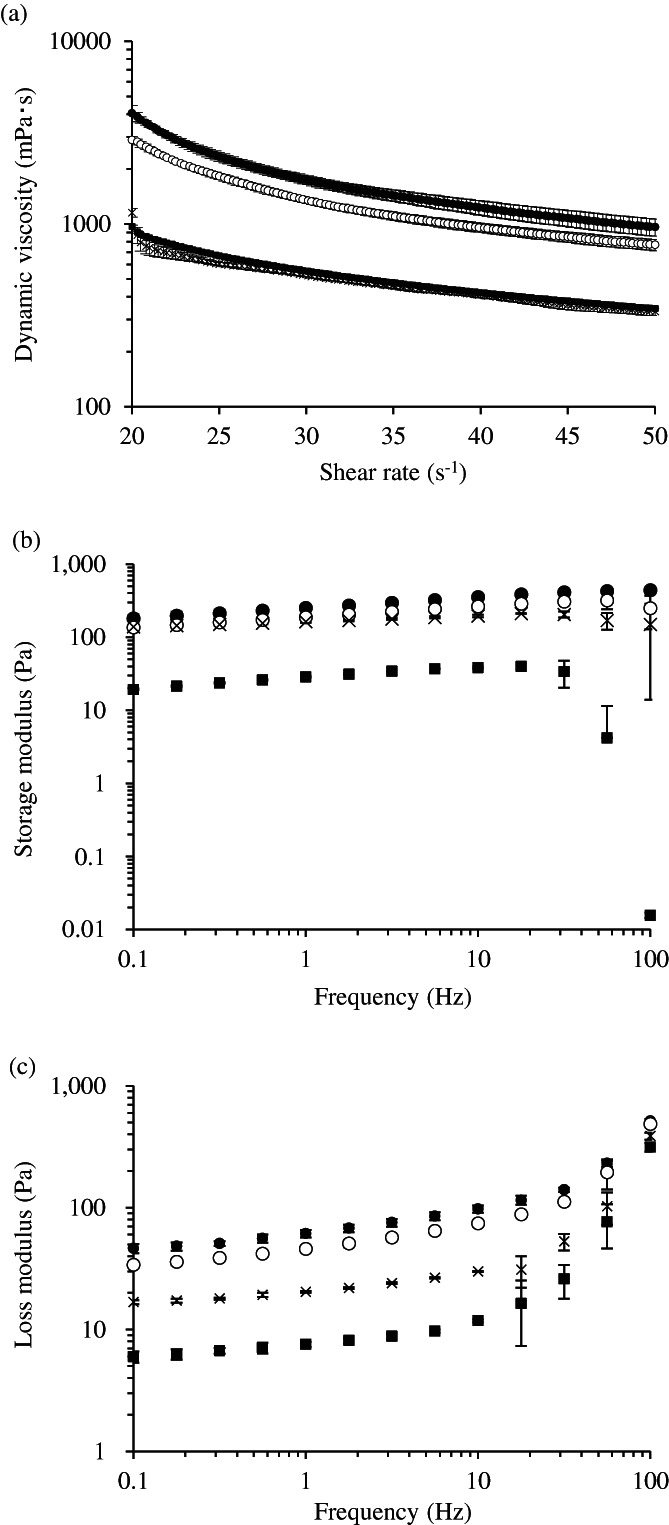
Measurements of rheological properties. Rheological properties of all samples were examined (*n* = 3). (a) change of dynamic viscosity by increasing shear rate, (b) frequency‐dependent storage modulus, *G'*, and (c) loss modulus, *G"*. The yogurt was tested at 10 and 20°C, and the other samples were tested at only 20°C. The symbols in the figure indicate: ● yogurt tested at 10°C, 〇 yogurt tested at 20°C, ■ food thickener tested at 20°C, and × deglutition aid jelly tested at 20°C

The adhesiveness of the yogurt, deglutition aid jelly, and food thickener were 81%, 31%, and 54%, respectively, in the glass beaker, and 91%, 11%, and 49%, respectively, in the PVP beaker (Figure 6). Adhesiveness of the deglutition aid jelly, which demonstrated lower effects on disintegration and dissolution of tablets than the other foods did, was low. To further examine physical properties, rheological measurements were performed (Figure 7). The viscosities of all the samples were inversely proportional to the shear rate. (Figure 7a). Therefore, it indicated that all samples had a shear‐thinning characteristic that is fluid at high shear rates. Besides, the viscosities of yogurt were higher than the viscosities of the other samples at all shear rates. The storage modulus (*G')* of yogurt and jelly ranged approximately from 100 to 400 Pa and was higher than that of the food thickener (Figure 7b). In addition, their values were slightly increased as frequency increased, while the values of yogurt (20°C) at 100 Hz and those of jelly at 31.6 Hz and 100 Hz subsequently decreased. The *G'* values of the food thickener increased marginally from 20 to 40 Pa as frequency increased, up to 17.8 Hz; after this, the values greatly decreased between 31.6 and 100 Hz. The loss modulus (*G")* of yogurt were highest, followed in order by those of jelly and the food thickener, and the *G"* values of all samples, especially the food thickener, were increased according to frequency increase (Figure 7c). The loss tangent (tanδ = *G”*/*G'*) values in yogurt and the food thickener were approximately 0.3, and the values in deglutition aid jelly were 0.1, between 0.1 and 10 Hz. Although they all exhibited gel‐like behavior, the strengths of the food thickener and yogurt were lower than that of the deglutition aid jelly. Additionally at high frequency regions, the tanδ value of all samples increased to >1, so they all indicated liquid‐like behavior. However, we should note that the measurement data might be unreliable due to the large standard deviation of the values between 10 and 100 Hz.

## 4 DISCUSSION

Yogurt is considered an easy‐to‐eat food for patients with dysphagia because of its appropriate viscosity and hardness. If yogurt can be used as an aid in taking oral medications, adherence would be expected to improve in patients who usually consume yogurt. It has been reported that yogurt prepared using the *L. cremoris* FC strain is better at preventing aspiration compared with yogurt made using other strains of lactic acid bacteria (Gotoh et al., 2019). In this study, we aimed to assess *L. cremoris* FC‐containing yogurt as a potential swallowing aid for oral tablets. The effects of *L. cremoris* FC‐containing yogurt on the disintegration of magnesium oxide tablets were low. During the dissolution experiment, drug release from yogurt‐immersed tablets was found to be lower, at 15 min, than that of non‐immersed tablets, but was similar to drug release of non‐immersed tablets or of those immersed in jelly and food thickener, at 120 min. The gastric retention times of medicines are generally within 120 min (Aulton & Taylor, 2013). Yogurt was found to delay the disintegration of some rapidly disintegrating tablets (certain magnesium oxide tablets and OD tablets). This tendency was similar to that of food thickeners, but the disintegration times tended to be slightly longer with yogurt than with the food thickener. Deglutition aid jelly, on the other hand, did not cause a delay in disintegration.

The adhesiveness of swallowing aid foods was in this order: yogurt > food thickener > deglutition aid jelly (Figure 6). We previously reported that the high adhesiveness of food thickener caused an elongation of the disintegration time of the tablets compared to low adhesiveness in food thickener (Matsuo et al., 2020). The results could demonstrate that deglutition aid jelly had a minimal effect on the tablets. Dynamic viscosity of the deglutition aid jelly was also lower than that of yogurt, but it was similar to that of the food thickener (Figure 7a). Deglutition aid jelly consists of solids and liquid (Figure 1). The solids exist as small boluses (Figure 1) and have stronger gel‐like behavior than yogurt and the food thickener (Figure 7b,c, tanδ). When the solids of the jelly attached to the tablets, voids of jelly solids around the tablets were formed, allowing water to easily invade the voids. It is thought that the minimal effects of deglutition aid jelly on disintegration and dissolution of tablets might be related to the solid and liquid nature of the deglutition aid jelly, not dynamic viscosity. In yogurt, the adhesiveness, dynamic viscosity, *G'*, and *G"* were the highest out of all the swallowing aid foods. Attachment of yogurt to the tablets might have occurred more easily, while detachment from the tablets might have been more difficult than those of the deglutition aid jelly and food thickener. Moreover, casein, which is found in yogurt, aggregates under acidic conditions (Niki, 2002). This might have caused the delay in disintegration and the reduction in dissolution rate in the acidic medium. However, our physical assessments of swallowing aid foods were performed at 10 and 20°C to obtain fundamental data for taking medications. After taking medications with swallowing aid foods, they move through the gastric duct, where they are sheared at a higher temperature of approximately 37°C. To further elucidate whether swallowing aid foods easily detach from tablets, further experiments at 37°C are warranted.

It is important to establish the safety of oral medications when taken with yogurt and to verify the effects of yogurts on various medications, both *in vitro* and *in vivo*. The pharmacological activities of bisphosphonates, tetracyclines, and quinolones are reduced by the calcium found in milk. Therefore, these medications cannot be administered with yogurt. Furthermore, the effects of yogurt on the taste of medications were not evaluated in this study. It is important to assess these factors to ensure medication adherence; this is an aspect which should be investigated in future studies. It is also crucial for pharmacists to confirm dietary habits and methods for taking medications in patients with dysphagia so that optimal swallowing aids can be recommended. Subsequent studies should investigate swallowing aids based on pharmacists' input on patient habits.

In conclusion, this study demonstrated that yogurt manufactured using *L. cremoris* FC did not remarkably affect the disintegration or dissolution profiles of magnesium oxide tablets or those of the other oral tablets evaluated. Similar to deglutition aid jelly and food thickeners, yogurt may be useful in helping patients with dysphagia take oral medications.

## CONFLICT OF INTEREST

Taisuke Matsuo was funded by Fujicco Co., Ltd. Yoshiyuki Tabata, Yayoi Gotoh, and Toshio Suzuki are employees of Fujicco Co., Ltd. The other authors declare no conflicts of interest.

## AUTHOR CONTRIBUTIONS

**Taisuke Matsuo:** Conceptualization (equal); formal analysis (lead); investigation (lead); project administration (lead); supervision (equal); writing – original draft (lead); writing – review and editing (lead). **Yoshiyuki Tabata:** Investigation (supporting). **Hina Sasaki:** Investigation (supporting). **Yuki Yoshida:** Investigation (supporting). **Yayoi Gotoh:** Investigation (supporting); writing – original draft (supporting); writing – review and editing (supporting). **Toshio Suzuki:** Conceptualization (equal); project administration (equal); writing – review and editing (supporting). **Michiko Obara:** Writing – review and editing (supporting). **Yasuyuki Sadzuka:** Writing – review and editing (supporting). **Takashi Tomita:** Conceptualization (equal); project administration (equal); supervision (supporting); writing – review and editing (supporting).

## ETHICAL STATEMENTS

Ethical Review: This study does not involve any human or animal testing.

## Data Availability

The data that support the findings of this study are available from the corresponding author upon reasonable request.
